# Public health implications of opening National Football League stadiums during the COVID-19 pandemic

**DOI:** 10.1073/pnas.2114226119

**Published:** 2022-03-22

**Authors:** Bernardo García Bulle, Dennis Shen, Devavrat Shah, Anette E. Hosoi

**Affiliations:** ^a^Institute for Data, Systems and Society, Massachusetts Institute of Technology, Cambridge, MA 02139;; ^b^Simons Institute for the Theory of Computing, Melvin Calvin Laboratory, University of California, Berkeley, CA 94720;; ^c^Department of Electrical Engineering and Computer Science, Massachusetts Institute of Technology, Cambridge, MA 02139;; ^d^Department of Mechanical Engineering, Massachusetts Institute of Technology, Cambridge, MA 02139

**Keywords:** COVID-19, synthetic control, sports stadiums, pandemic policies

## Abstract

Using attendance data from the 2020 National Football League (NFL) regular season and local COVID-19 case counts, we estimate the public health impact of opening NFL stadiums to fans during the COVID-19 pandemic. Data are analyzed using robust synthetic control, a statistical method that is employed to obtain counterfactual estimates from observational data. Unlike previous studies [J. Kurland et al., SSRN, 2021], which do not consider confounding factors such as evolving policy landscapes in different states, the synthetic control methodology allows us to account for effects that are county specific and may be changing over time. We find it is likely that opening stadiums had no impact on local COVID-19 case counts; this suggests that, for the 2020 NFL season, the benefits of providing a tightly controlled outdoor spectating environment—including masking and distancing requirements—counterbalanced the risks associated with opening. These results are specific to the 2020 NFL season, and care should be taken in generalizing our conclusions. In particular, 1) these data reflect a period during which earlier strains of COVID-19 were dominant prior to the emergence of more-transmissive strains such as the Delta and Omicron variants, and 2) the data are restricted to outdoor environments; hence our results cannot be applied to small indoor spaces where transmission-restricting controls are essential.

## Significance

Using data from 2020, we measure the public health impact of allowing fans into sports stadiums during the COVID-19 pandemic; these results may inform future policy decisions regarding large outdoor gatherings during public health crises. Second, we demonstrate the utility of robust synthetic control in this context. Synthetic control and other statistical approaches may be used to exploit the underlying low-dimensional structure of the COVID-19 data and serve as useful instruments in analyzing the impact of mitigation strategies adopted by different communities. As with all statistical methods, reliable outcomes depend on proper implementation strategies and well-established robustness tests; in the absence of these safeguards, these statistical methods are likely to produce specious or misleading conclusions.

A year and a half into the global COVID-19 pandemic, we have an opportunity to analyze and reflect upon the policies and decisions enacted over the past 18 mo. Given the distributed nature of policy decisions in the United States, we find ourselves in a unique position in which states and municipalities have explored different strategies to combat the virus, and the efficacy of those policies has been imprinted in the local case counts, hospitalizations, and death records. In particular, these data contain a wealth of information about which policies have proven to be effective in preserving the health and safety of our communities.

One activity that one may wish to consider is the opening of outdoor sporting events to spectators. This question has recently generated quite a bit of interest as ballparks across the nation open for summer and events such as the 2021 Summer Olympics in Japan take place.* On the one hand, governing bodies are naturally wary of opening stadiums given the well-documented importance of avoiding large gatherings. On the other hand, sporting events are often held outdoors, where airflow is largely unobstructed (1), and in venues where crowd density can be carefully controlled if the event is properly managed. In the absence of a detailed analysis, it is not immediately obvious which of these effects dominates.

Data from the National Football League (NFL) may provide an answer to this question. During the 2020 regular season, teams in the NFL collaborated with local communities to determine whether or not to allow fans in the stadiums during the pandemic. In general, stadiums that opened their doors to fans adopted pandemic requirements for all in attendance (1), which typically include some combination of staggered entry, required masking, health questionnaires, temperature checks for staff, deployment of compliance officers, modified concessions, social distancing in seating and lines, mobile ticketing, enhanced cleaning protocols, amplified health and safety communications, and capacity limitations. The highest capacity that any NFL stadium allowed during the 2020 regular season was 30% (Dallas), with most other stadiums considerably below that limit (2). These policy decisions were made based on local guidelines, local prevalence, community risk tolerance, and other localized considerations; some stadiums ultimately decided to allow fans at the games, while others remained closed, providing perhaps the first set of natural experiments that can be analyzed to investigate the impact of opening stadiums on COVID-19 case rates. In the words of Kurland et al. (3), who recently provided a first look at this data, “Scant evidence has been gathered in the extant literature on the impact of sport venues on local public health, influenza-related mortality rates, or disease contagion more generally. There is a complete absence of any evidence related to the impact of fans gathering at sporting events, or mass gatherings more generally, on incidence of COVID-19 at the local-level.” The natural experiments from the 2020 NFL season and other sports leagues present a golden opportunity to address these questions in the context of the original 2020 COVID-19 strain (4, 5).

In the Kurland et al. (3) study, the authors compared COVID-19 case data from NFL stadium counties that allowed fans in the stadium to counties that did not allow fans, and looked for spikes in the data in the weeks following a game; the authors concluded, from this analysis, that the presence of large numbers of fans at NFL games led to “tangible increases” in the local incidence of COVID-19 cases. However, this type of analysis may be problematic: In this context, the control stadiums (i.e., those without fans) tend to be embedded in states with stricter COVID-19 policies—rather than a random control—so the sample of control counties is strongly biased. New York and Dallas, for example, are immersed in very different environments with different pandemic policies, and it is not at all obvious that one can attribute the differences in case spikes to the stadiums, given the enormous number of confounding factors.

Fortunately, there exists a rich literature of techniques—longitudinal methods, hierarchical methods, factor model methods, synthetic control, etc.—that we can draw upon to account for these confounding factors. In this particular analysis, we turn to synthetic control (6–9), which has been applied in a diversity of fields—criminology (10), healthcare (11), sports (12), and political science and policy evaluation (13–15), to name a few. At its heart, synthetic control is a method for estimating a counterfactual in the absence of an intervention, in this case, what would have happened if stadiums had not opened. The method provides a systematic way to choose relevant comparison units when randomized controls are not available.

To illustrate the power of synthetic control, imagine the ideal experiment one would like to run in order to quantify the impact of opening the Dallas stadium to fans. In principle, we would like to have COVID-19 case counts from Dallas County throughout the season with the stadium open to fans and case counts from a Dallas twin—with identical people and policies to the first Dallas—in which the stadium did not open for comparison. The first set of data (Dallas open to fans) is readily available. The second set of data can be constructed from information from other counties in Texas—hereafter referred to as donor counties—which have policies and characteristics similar to Dallas. Synthetic control provides a methodology to build a weighted combination of these Dallas-like counties, which can then be used as a control group, that is, a “synthetic” Dallas twin. In particular, we seek the linear combination of case counts from other Texas counties that most closely mirrors the Dallas case counts prior to the stadium opening. Given that none of these non-Dallas counties have a stadium, this linear combination can be extended postintervention (i.e., after opening the stadium) to estimate what would have happened in the synthetic Dallas in which no stadium opened. Once it has been established that the stadium county and the synthetically generated county have similar behavior over extended periods of time prior to the intervention, a discrepancy in the number of COVID-19 cases following the intervention may be interpreted as a result of allowing fans in the stadium. One of the advantages of this method is that it can account for the effects of confounding factors that are county specific and may be changing over time, which is crucial in the ever-evolving policy landscape of a pandemic (16). In particular, our methodology allows for correlation between the decision to open the stadium and characteristics that define the county (cultural or political leaning, population density, demographics, etc.), but cannot account for correlations between the decision and exogenous noise.

At this point, it is reasonable to speculate whether one should expect linear combinations of donor counties to accurately represent stadium counties (both observed and counterfactual). In general, assuming linearity is appropriate provided there exists an underlying low-dimensional structure to the case count data, that is, if the matrix containing discretized time series of donor county case counts is approximately low rank. Under a such a setting, linearity between counties is an almost immediate consequence (see *Materials and Methods* for details). This low-rank assumption is common in the matrix completion literature; notably, low-rank matrices have also been shown to naturally arise in modern datasets and emerge from “well-behaved” generative models (e.g., Lipschitz functions) (17–20). This point will be revisited in *Results*, where we test for low rankedness empirically in the context of our dataset.

Finally, the selection of donor units is a critical step in the successful implementation of creating a synthetic control. In particular, donor units (in our case, counties) should have the following characteristics:

1) Counties affected by the intervention or by events of a similar nature should be excluded from the donor pool.
2) Counties that may have suffered large “idiosyncratic shocks” (7, 21) during the preintervention period should be excluded.
3) The donor pool should be restricted to counties with characteristics similar to the stadium county; in this case, we restrict our pool to counties from the same state to maintain some consistency in COVID-19 policies.
4) Case counts that cover an extended period of time prior to the intervention are required for both stadium counties and donor counties.

In order to establish which counties satisfy these constraints, the NFL provided us with aggregate attendance data indicating the percentage of fans from each county in each state (2). In general, 10% or more of the fans come from the county in which the stadium is located. Hence, we designate counties that provided more than 10% of the fan base as stadium counties. In addition, there are a number of counties that are home to many fans but not to the same extent as that of the stadium counties. Since there is some ambiguity as to whether these counties should be counted as stadium counties or donor counties, we designate counties that supply between 1% and 10% of the fan base as buffer counties and, in light of the first criterion above, do not include them as either stadium or donor counties. Second, to address criterion 3, we only include counties in the donor pool that come from the same state as the stadium county. Although there is variation at the county level, overarching COVID-19 guidance, in general, comes from the states; hence, we assume that policies are relatively consistent within states and allow that they may vary dramatically from state to state. In addition, we only retain counties in which at least 200 cases have been recorded, in order to eliminate donor counties that are either markedly underreporting or undertesting. Finally, we are fortunate that football season starts in September, which allows us to address criterion 4; given that relatively reliable COVID-19 case count data have been available since approximately April 2020, we have 4 mo of training data at our disposal to learn the weights for the synthetic counties. Criterion 2 is trickier, given that we do not necessarily know, a priori, all events that could cause a shock to the system; however, a posteriori, we can investigate the outcomes and look for signs of such a shock.^†^

## Results

Using measured county-level COVID-19 case data (22), synthetic counties were constructed for all NFL stadium counties except for Maricopa County, home of the Arizona Cardinals. Unlike the other stadiums, fan origin county data were not available for Maricopa; hence the Cardinals were omitted from our study. In all cases, rather than considering individual games, which rapidly becomes murky given that the long-term impact of one game may overlap with the next, we simply identify stadiums as open or closed for the season starting with the first game in which fans were allowed in the stadium. COVID-19 case counts in the synthetically constructed counties were then generated and compared to measured case counts. Precisely, we define the difference between the synthetic county and the measured county as

[1] $$\Delta (t)=c(t)-c_{\text{synth}}(t),$$

where *c*(*t*) is the cumulative number of reported COVID-19 cases in the stadium county, and $c_{\text{synth}(t)}$ represents the counterfactual number of cases in the synthetic county, that is, the number of cases one would expect if the stadium remained closed (see *Materials and Methods*). Positive Δs indicate excess cases in stadium counties; negative Δs indicate fewer than expected cases in stadium counties relative to the counterfactual.

**Fig. 1.**
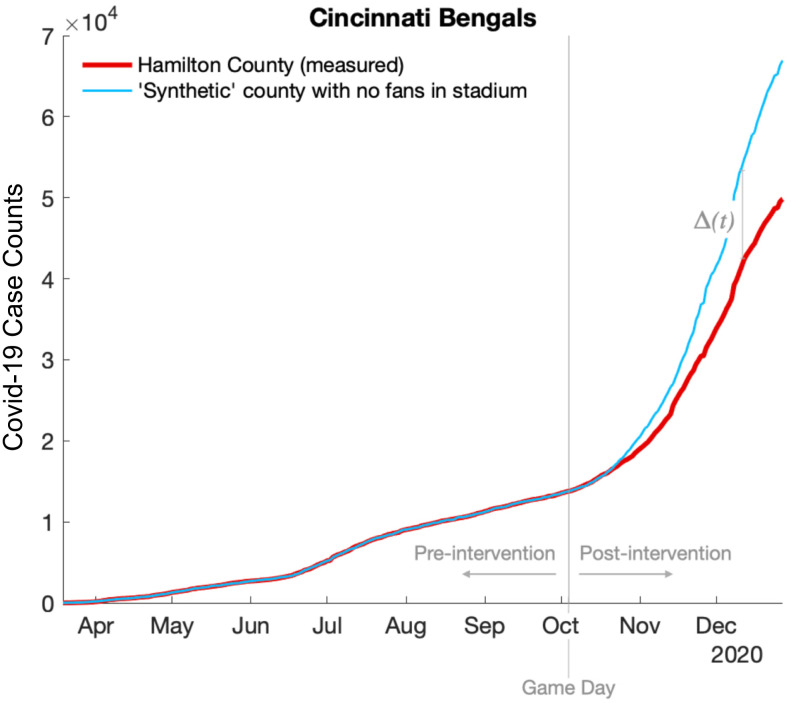
Comparison of the measured COVID-19 case counts from Hamilton County, OH (red line), and COVID-19 case counts from the counterfactual synthetic county (blue line). The vertical gray line indicates the date of the first home game that allowed fans in the stadium. In this example, the stadium county recorded fewer cases than the counterfactual after fans were allowed in the stadium, suggesting that, for Hamilton County, the benefit of moving fans into a controlled outdoor environment outweighed the potential harm associated with large gatherings.

A sample county—Hamilton, OH, home of the Cincinnati Bengals—is shown in Fig. 1. Prior to the intervention (i.e., opening the stadium), the measured COVID-19 case counts (red line) and the case counts from the synthetic county (blue line) are indistinguishable. Given the physiological characteristics of the virus, we should not expect the impact of the intervention to appear in the case count data until 1 wk to 2 wk after the event. This is indeed the case for Hamilton County, where the real data continue to closely track the measured date for ∼14 d. Following this 14-d period, measured case counts begin to deviate from the counterfactual; interestingly, the measured counts are slightly lower than the projected counts, suggesting that, in this particular county, opening the stadium may have modified fan behavior in a way that was helpful to the community, and not harmful.

**Fig. 2.**
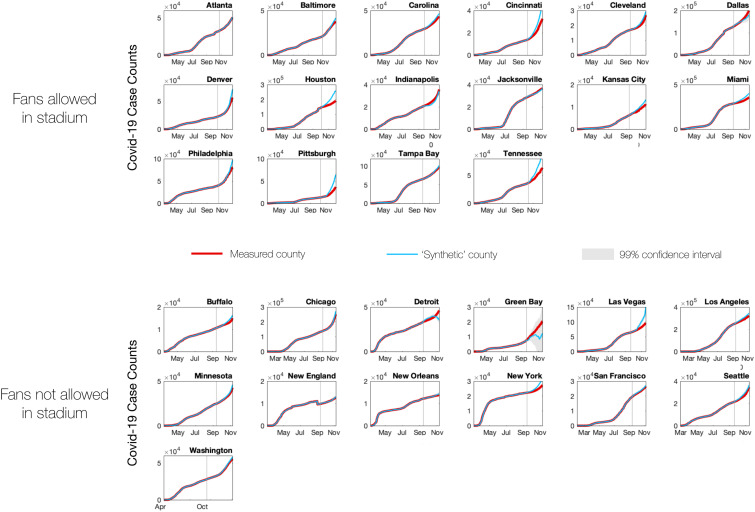
Comparison of measured case counts with synthetic county case counts for all NFL stadium counties except Maricopa County. The top 16 plots show stadiums that allowed fans for some portion of the 2020 season; the bottom 13 plots show stadiums that remained closed. Red lines indicate measured data; blue lines indicate synthetic data; light gray shaded regions indicate 99% prediction intervals; vertical gray line indicates the first day that the stadium was open to fans (for open stadiums) or the date of the first home game (for closed stadiums).

Similar plots for all NFL stadium counties are shown in Fig. 2. In addition to considering case counts in stadiums that opened to fans, we also computed expected case counts in stadium counties that did not open to fans. If the synthetic control approach is performing properly, we should not see any difference between the synthetic control counties and the measured data in the counties where stadiums remained closed to fans. As expected, on average, stadiums without fans show no significant difference from the synthetic counties.

## Discussion

### Impact of Opening Stadiums to Fans.

**Fig. 3.**
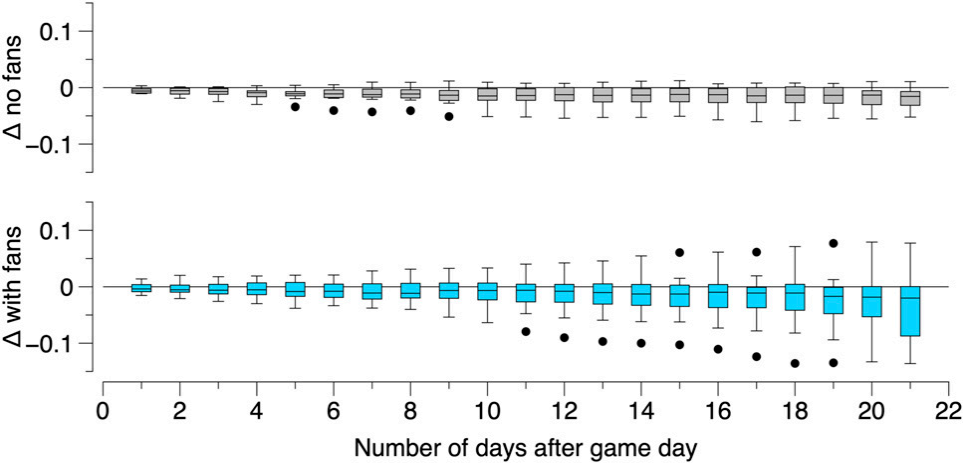
(*Top*) Gray IQR box-and-whiskers plots showing the difference between measured case counts and synthetic case counts, Δ(t), up to 21 d after the first home game for stadiums that did not open to fans. If the synthetic approach is working reliably, the gray box-and-whisker points should be indistinguishable from zero, given that no fans were allowed in the stadium, and hence there was no intervention. (*Bottom*) Blue IQR box-and-whiskers plots showing the difference between measured case counts and synthetic case counts, Δ(t), up to 21 d after the first game for counties with stadiums that opened during the pandemic. Again, the points show no significant difference from zero, indicating that allowing fans in the stadium had no impact on the local prevalence of COVID-19. Note that negative Δs signify fewer cases in stadium counties relative to the counterfactual.

The most remarkable feature of our results is how unremarkable they are. By and large, the synthetic counties are well behaved (exceptions will be discussed below), and the analysis shows no indication that opening stadiums had any impact on community spread. In contrast to the Kurland et al. (3) study, we find that counties which allowed fans in the stadium show no statistically significant difference from the synthetic counties; that is, there is no evidence that the NFL’s controlled opening of stadiums to fans led to any increase in COVID-19 cases. Fig. 3 shows the interquartile range (IQR) difference between synthetic case counts and measured case counts for stadiums that opened to fans (blue) and those that did not (gray). Neither are statistically different from zero, although data from stadiums with fans show a longer tail skewed toward a negative Δ, hinting that providing controlled outdoor environments for fans to assemble may have benefited some counties.

### Large versus Small Crowds.

**Fig. 4.**
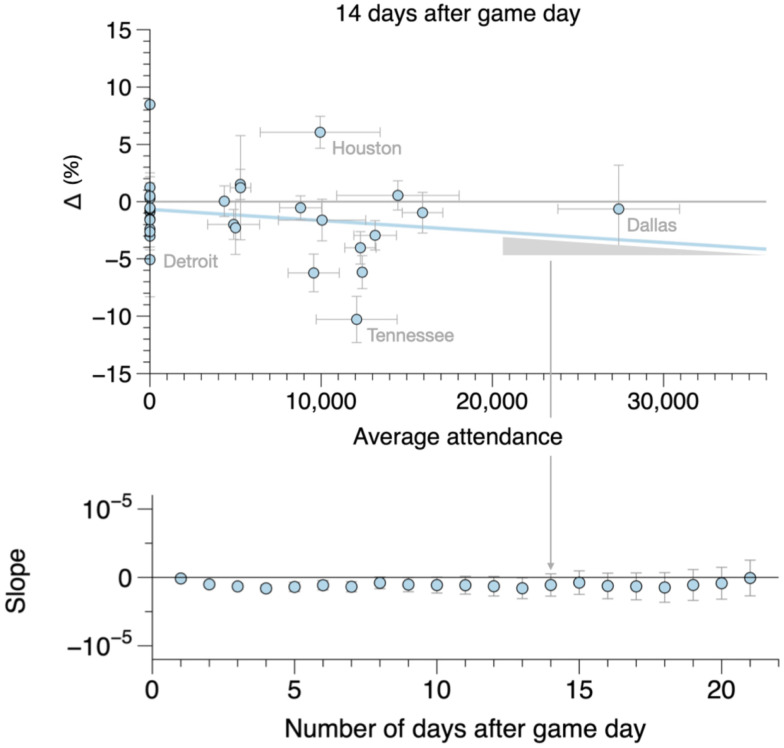
(*Top*) Difference (by percentage) between stadium and synthetic counties 14 d after the stadium first opened to fans versus average attendance. Negative Δs indicate counties in which the measured case counts were lower compared to the counterfactual. The blue line is a linear fit. (*Bottom*) Slope of the linear regression versus number of days after game day. Data indicate that there is no correlation between attendance and COVID-19 case counts.

Given that most stadiums were operating far under their capacity limits, one might argue that the null result above is dominated by stadiums with small attendance numbers, which may overshadow the signal from stadiums that allowed more fans to attend games. In order to address this, we investigated the impact of the number of fans in attendance on community spread. Fig. 4 shows the difference between case counts of the synthetic counties and measured case counts 14 d after the stadium opened to fans, Δ(t=14), as a function of the number of fans in attendance. To determine whether there is any correlation with attendance numbers, a linear regression was performed for each day following the intervention in the subsequent 3 wk. The measured slopes from these regressions are also shown in Fig. 4. Again, contrary to Kurland et al. (3), our analysis shows no correlation with attendance. Stadium counties that allowed higher attendance show no increase in COVID-19 cases relative to their lower-attendance counterparts or to stadiums that did not open to fans at all.

These two null results are perhaps unsurprising, as the relevant comparison is not necessarily fans at stadiums versus fans isolated at home. Rather, we need to consider what the fans would have been doing (23) had they not been at the game. While these details are purely speculative—for example, fans may be home, or they may be at a bar watching the game, or they may be watching the game indoors with friends—synthetic control obviates the need for speculation. In this case, the data suggest that having fans outdoors at the stadium in a controlled environment—that is, with face coverings, in family pods, socially distanced—is no worse than what they would do otherwise. For the original strain in the 2020 phase of the pandemic, it was well established that interacting outdoors is better than indoors (24); hence, spreading people out in a large outdoor stadium may be preferable to the alternatives. Although we may not know precisely what these alternatives are, synthetic control provides a mechanism to estimate the impact of the most realistic alternatives based on measured human behavior.

### Validity of the Synthetic Control Approach.

**Fig. 5.**
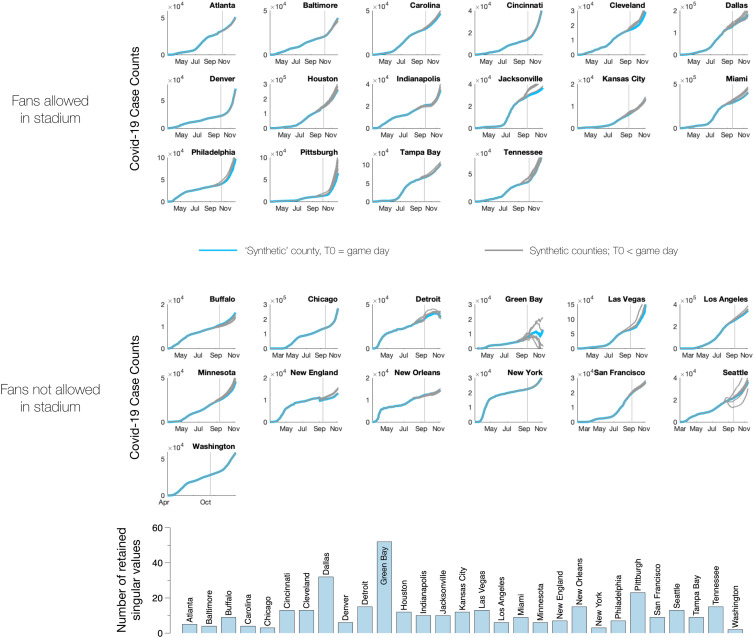
(*Top* and *Middle*) Blue lines indicate synthetic case counts computed using the entirety of the preintervention period. Gray lines indicate synthetic case counts computed using a subset of the preintervention data, that is, assuming the intervention happened 1 wk to 6 wk prior to game day. The majority of the plots show little dependence on the intervention date suggesting that the synthetic counties in those cases are robust; others—for example, Green Bay, Seattle, and Jacksonville—indicate that the synthetic counterfactual is not reliable for those counties. (*Bottom*) Number of singular values in the donor matrix that are retained to construct each synthetic stadium county. By and large, a low-dimensional representation suffices (again, with a few notable exceptions such as Green Bay).

As is the case with all statistical methods in generating counterfactuals, a considerable amount of care must be exercised in applying the synthetic control approach. In particular, there are a number of variants of the originally proposed synthetic control estimator of refs. 6 and 7, as well as robustness tests that can be applied to check the validity of our synthetically constructed counties. In this work, we adopt the estimator proposed in ref. 25 and follow the tests laid out in refs. 16 and 21. First, given that we have an abundance of donor counties, there is a danger of overfitting the preintervention period. To guard against this, we apply a principal component regression (PCR) analysis and project our donor counties onto a low-dimensional subspace that has been constructed by retaining only the largest singular values in our donor matrix (see *Materials and Methods*). In the context of synthetic controls literature, this variation of the original method is also called robust synthetic control. The number of singular values that are retained is determined by the fit in the preintervention period; here we require that the synthetic county case counts and the measured county case counts differ by less than 1% in aggregate during the preintervention period. If the synthetic case counts are well represented in our low-dimensional space, we can have some confidence that they are reflective of a real signal rather than overfitting noise; in most counties, ∼10 or fewer singular values are sufficient to capture the variance in the preintervention period which typically consists of a few hundred data points, that is, one point per day in the months prior to opening (Fig. 5, *Bottom*). Furthermore, if the *R*^2^ value in the preintervention period is large (in our case $R^{2}>0.99$ ), then the target latent factor is likely well represented within the space spanned by the donor units’ latent factors, suggesting that linear combinations of the donors suffice to eliminate confounders. The number of singular values that are retained in each stadium county are shown in Fig. 5; with the exception of Green Bay, Dallas, and Pittsburgh, we find that a low-dimensional ( <20) representation suffices to capture preintervention dynamics.

A second test for robustness of counterfactual estimates is to vary the intervention date *T*_0_. If the synthetic construction is robust, it should not depend sensitively on *T*_0_, provided that the fitting period occurs prior to the intervention. To test this, we compared our synthetic county case counts, which were computed using the entirety of the preintervention period $\left[0,T_{0}\right]$, with synthetic county case counts constructed using the interval $\left[0,T_{0}-\tau \right]$. Results for τ= 1, 2, 3, 4, 5, and 6 wk are shown in Fig. 5. Changing *τ* had little effect on most counties, with the exception of (again) Green Bay, Jacksonville, and Seattle.

**Fig. 6.**
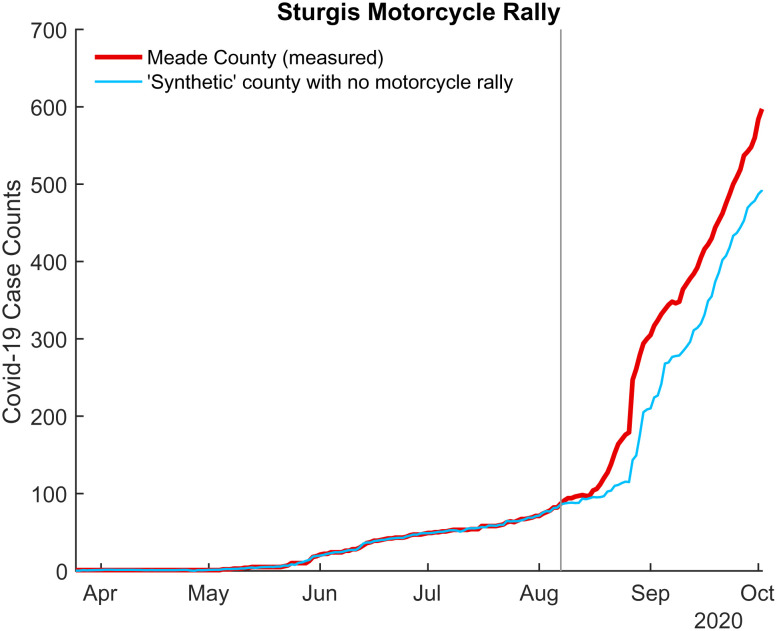
Comparison of measured COVID-19 case counts (red line) and COVID-19 case counts from a counterfactual synthetic Meade County (blue line) for the Sturgis Motorcycle Rally. The vertical gray line indicates the first day of the rally.

Third, given that we are reporting a null result, it behooves us to investigate whether our synthetic control methodology has sufficient sensitivity to capture an increase in COVID-19 cases following a known spreading event. To test this, we considered data from the Sturgis Motorcycle Rally which took place in mid-August in 2020. The Sturgis Motorcycle Rally is widely believed to have been a superspreading event, as revealed in a working paper by Dave et al. (26) in which the authors used anonymized cell phone data from SafeGraph, Inc. to identify origin counties of rally goers. Using these data, we generated a synthetic Meade County using counties with moderately low and low inflow attendance (as defined in Dave et al.) as donor counties. Synthetic case counts are compared to measured case counts in Fig. 6. The synthetic control approach does indeed find a significant increase in COVID-19 cases in Meade County following the rally and suggests that Sturgis may be responsible for a 24%±11% increase in COVID-19 cases after 14 d and a 43%±11% increase after 21 d.

### Connection to Other Observed Variables.

The fourth and final consistency check we apply is to examine whether counties that are “close” to one another in our low-dimensional representation are also “close” with respect to relevant observed variables. For example, given the high level of politicization around COVID-19 policies (masking, distancing, etc.), one might expect counties with similar COVID-19 profiles to also share similar political views. To test this hypothesis, we compute the distance in our low-dimensional subspace of each county, *i*, from the most Democratic county, $d_{D,i}$, and from the most Republican county, $d_{R,i}$, in the state (see *Materials and Methods*). Note that these are not geographic distances; rather, they are distances defined in the subspace constructed from COVID-19 case counts. Hence, two counties are “close” if they share similar temporal COVID-19 case profiles. These two distances are then combined into a single metric for the *i*th county,

[2] $$P_{i}=\frac{d_{R,i}}{d_{R,i}+d_{D,i}}.$$

**Fig. 7.**
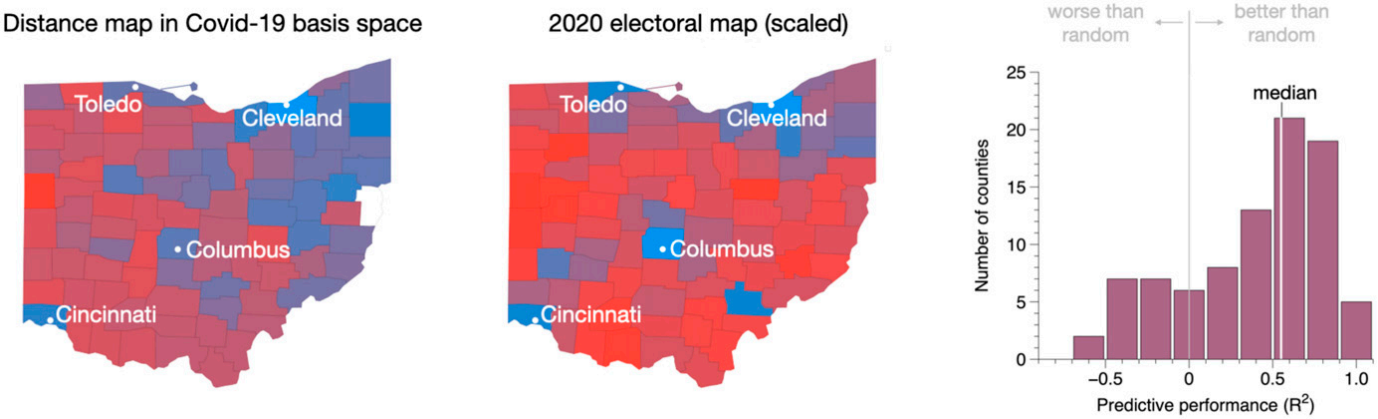
(*Left*) Map of Ohio colored by *P_i_*, the relative distance defined in the subspace constructed from COVID-19 case counts, from the most Democratic county in the state (Cuyahoga) and the most Republican county (Mercer). (*Middle*) The 2020 electoral map of Ohio. Colors have been scaled in both maps such that Cuyahoga (blue) and Mercer (red) represent the extremes of the color scale. (*Right*) Histogram of counties depicting the predictive power of the COVID-19 subspace; 1 = perfect estimator; 0 = no better than random; median = 0.55.

Thus, $P_{i}\approx 0$ for counties with temporal profiles similar to the most Republican county, and $P_{i}\approx 1$ for counties similar to the most Democratic county. A sample state (Ohio) in which counties have been colored by *P_i_*s is shown in Fig. 7, along with the corresponding 2020 electoral map. Both maps have been scaled such that the most Democratic county corresponds to one (blue) and the most Republican county corresponds to zero (red).

Remarkably, distances in the COVID-19 generated subspace do not simply follow geographic trends; rather, they highlight Cuyahoga County (Cleveland), Hamilton County (Cincinnati), Franklin County (Columbus), Lucas County (Toledo), and the upper east side of the state as similar, mirroring the electoral map of the state. To estimate the extent to which variance in voting patterns is captured in the temporal COVID-19 signature, we define a pertinent *R*-squared for the *i*th county,

[3] $$R_{i}^{2}=1-\frac{\epsilon_{\text{covid},i}}{\epsilon_{\text{rnd},i}},$$

where the difference between the predicted and measured *P_i_*, namely, $\epsilon_{\text{covid},i}\equiv |P_{i}-P_{\text{meas},i}|$, has been normalized by the difference one would expect from a randomly generated estimator $P_{\text{rnd},i}$ (see *Materials and Methods* for details). A histogram of $R_{i}^{2}$s for Ohio is shown in Fig. 7, which reveals a median *R*^2^ of 0.55. Performing a similar exercise with population and geographic distance from Cuyahoga and Mercer Counties—with geographic distance defined analogously to the political distance *P_i_* in Eq. 2—we find *R*^2^s of 0.36 and 0.29, respectively. Given that the focus of this study is on the impact of opening stadiums, we will refrain from digging more deeply into the implications of this mapping exercise, and merely emphasize that our low-dimensional COVID-19 subspace is indeed reflective of other pertinent observed variables—geography, population, and political leaning—as one might expect. It is intriguing to note that, in this particular state, voting patterns show the strongest connection to COVID-19 temporal signatures, suggesting a potentially promising avenue for future study.

Finally, given the above relationship between temporal case profiles and political leaning, and the fact that stadiums tend to be located in urban centers, one could imagine that there may be instances where a stadium county does not lie in the subspace spanned by the donor counties (e.g., if Cuyahoga County is politically blue and the rest of Ohio is red, we are left with the potentially dubious task of reconstructing a blue county from a linear combination of red ones). If this occurs, there is a danger that the synthetic counties (which are evolving as red counties) may become less reflective of their real counterparts (which are evolving as blue counties) as the pandemic and concomitant mitigation strategies evolve. In general, if the changes in mitigation measures are captured through latent confounders that factorize, then our method is robust against this scenario. To determine whether this is indeed the case, we can check whether the divergence between synthetic and real counties correlates with the difference between the political leaning of the stadium county and the political leaning of the state (i.e., the degree to which the stadium county is an outlier). We find that the difference in political leaning between the stadium county and the average donor county is always positive, confirming that stadium counties are bluer than donor counties, on average; however, we also find that there is no correlation between Δ(t) and outlier status, suggesting that the above-average “blueness” of stadium counties does not bias the results and that there are a sufficient number of stadium-like counties in the donor set to capture the impact of evolving mitigation strategies (as one might expect if, e.g., the mitigation landscape is dominated by state-wide—rather than local—policies).^‡^

### The Trouble with Green Bay.

At this point, it is evident that, although most synthetic stadium counties pass the consistency tests described above, there are a few that are problematic. In those counties, the failure of the synthetic control approach suggests that one of the four donor county criteria have been violated or that there is a problem with the county data. To determine the underlying cause of these failures, we take a brief deeper dive into the most egregious example: the Green Bay Packers. Given that the case counts are cumulative, there is clearly a problem with the Green Bay synthetic county data, which do not increase monotonically. In addition, Green Bay presents neither a satisfactory low-dimensional representation (requiring 52 basis functions to capture the preintervention period) nor a lack of sensitivity to the boundaries of the preintervention interval. A visual inspection of the donor county case counts reveals that an anomalous increase in cases occurred in a large subset of counties approximately 2 wk before the first NFL home game, and, upon further investigation, we find that this anomaly was due to a reporting error at the Wisconsin Department of Health Services (27). This error in reporting violates the condition that donor counties should not suffer a large idiosyncratic shock in the preintervention period and provides additional assurance that the method is capable of flagging prominent issues in the case data.

## Conclusions

In conclusion, we find no evidence that opening NFL stadiums to fans during the 2020 regular season led to any uptick in the number of COVID-19 cases in the stadium counties. Furthermore, our results highlight the fact that the policy environment in which counties are embedded is nonnegligible; hence, cross-state comparisons may be suspect if these environmental factors are not taken into account (3). While this study indicates that the measures taken by the NFL to open stadiums safely in 2020—including required masking, social distancing, amplified health and safety communications, and capacity limitations—were successful, it is important to recognize that the 2020 season took place before the B.1.1.7, Delta, and Omicron variants gained a foothold in the United States. All three variants are known to be significantly more transmissive than the original strain. As such, a similar analysis following the 2021 baseball and American football seasons and other global sports seasons are worth pursuing in order to guide sound policy decisions.

## Materials and Methods

### Causal Framework.

Here we utilize the causal framework of ref. 16. Specifically, it follows from the Neyman–Rubin causal model (28, 29), where the potential outcome for county *i* in period *t* is denoted by $Y_{it}^{\left(d\right)}$, with d∈{0,1} corresponding to the exposure to a binary treatment of opening or closing a stadium. Let *d* = 0 represent a closed stadium (i.e., control) and *d* = 1 represent an opened stadium (i.e., treatment). We denote $D_{i}\in \{0,1\}$ as the treatment assignment for county *i*. In line with the standard assumptions in the causal inference literature, we assume SUTVA (Stable Unit Treatment Value Assumption); namely, that our outcomes satisfy $c_{i}(t)=D_{i}\cdot Y_{it}^{\left((1)\right)}+(1-D_{i})\cdot Y_{it}^{\left(0\right)}$. Below, we highlight a few aspects of our causal framework to justify our algorithmic approach in estimating Eq. **1**, where $c_{i,\text{synth}}(t)$ denotes our estimate of $Y_{it}^{\left(0\right)}$ (in expectation); namely, we discuss the types of confounding that are allowed, and argue that a linear predictive model is appropriate for counterfactual estimation.

### Key Assumptions.

**Fig. 8.**
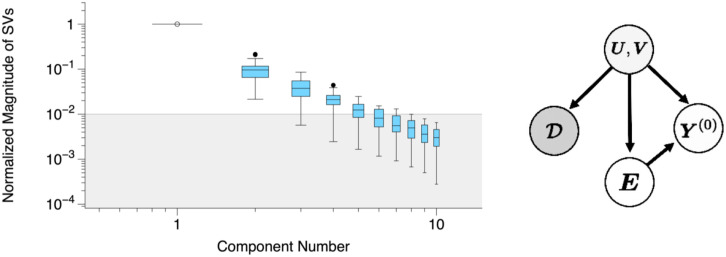
(*Left*) First 10 singular values of Φ; box and whiskers represent the range of values measured over all stadium states. Gray region indicates values that are less than 1% of the first component; most component numbers above 6 fall in this gray region, suggesting that a low-rank assumption is reasonable. (*Right*) Causal directed acyclic graph describing the type of confounding allowed in this work. Here, U,V are the latent factors that describe the characteristics of counties and time periods, E are the idiosyncratic shocks, $Y^{\left(0\right)}$ are the potential outcomes under control, and D are the treatment assignments.

Our potential outcomes under control $\left\{Y_{it}^{\left(0\right)}\right\}$ are encoded into a matrix $Y^{\left(0\right)}\in R^{N\times T}$, where *N* and *T* represent the total number of counties and time periods of interest, respectively. Our aim is to recover $\left\{Y_{it}^{\left(0\right)}:\forall i\text{s}.\text{t}.D_{i}=1\right\}$, which corresponds to the missing entries in $Y^{\left(0\right)}$. In order to recover these missing values, we make the following assumptions. First, we posit $Y^{\left(0\right)}=UV^{T}+E$, where $U\in R^{N\times r}$ and $V\in R^{T\times r}$ represent the matrix of latent factors associated with the counties and time periods, respectively, with r≪min{N,T}, and $E\in R^{N\times T}$ represents zero-mean idiosyncratic shocks. It follows that $E[Y^{\left(0\right)}]=UV^{T}$; we note that such a form always exists, by the singular value decomposition (SVD). Thus, our key assumption is that $E[Y^{\left(0\right)}]$ is a low-rank matrix, which is a standard assumption in the matrix completion literature. Evidence of low rankness can also be verified empirically (to an extent), and is shown in Figs. 5 and 8; Fig. 8 presents the magnitude of the 10 largest singular values for our data.

To characterize the types of confounding that are allowed, let $D=\{D_{i}\}$ denote the set of treatment assignments across all *N* counties. We posit that D⊥E | U,V, as shown graphically in Fig. 8, *Right*; coupled with our structural assumption, this implies that $D⊥Y^{\left(0\right)}\;|\;U,V$. In words, our assumption allows for the decision to open a stadium to be influenced by (i.e., correlated with) the characteristics that define the county (e.g., cultural or political leanings, population density, and demographics) and time period; these characteristics can also drive the underlying expected potential outcomes, as illustrated in Fig. 7. However, the decision cannot be impacted by exogenous noise.^§^

Finally, we discuss the implications of these two assumptions along with SUTVA. To begin, we note that, under a low-rank setting, linearity between counties is an almost immediate consequence and holds with high probability (w.h.p.). To see this, suppose our interest is in a treated county *i* (i.e., *D_i_*  =  1). If $\text{span}(\{u_{\ell }:D_{\ell }=0\})=R^{r}$, where $u_{\ell }$ denotes the ℓth row of ***U*** corresponding to the latent factor of county ℓ; then, it follows that $u_{i}\in \text{span}(\{u_{\ell }:D_{\ell }=0\})$, since $u_{i}\in R^{r}$; that is, $u_{i}=\sum_{\ell :D_{\ell }=0}\alpha_{\ell }u_{\ell }$ for some vector *α*. More generally, if the rows of ***U*** are randomly sampled Gaussian vectors, then $\text{span}(\{u_{\ell }:\ell \in S\})=R^{r}$ for any set S holds w.h.p., provided |S|≥r is chosen to be sufficiently large; see ref. 31 for details. This linearity implication that follows from low rankness, along with our assumptions, leads to the following identification result (17): conditioned on {D,U,V},

[4] $$\begin{array}{ll} E[Y_{it}^{\left(0\right)}\;|\;u_{i},v_{t}] & =\sum_{\ell :D_{\ell }=0}\alpha_{\ell }E[Y_{\ell t}^{\left(0\right)}\;|\;D,U,V]\\ \; & =\sum_{\ell :D_{\ell }=0}\alpha_{\ell }E[c_{\ell }(t)\;|\;D,U,V], \end{array}$$

where the final equality uses SUTVA. Namely, Eq. **4** states that, for any time period *t*, the expected potential outcome for county *i*, had it not opened its stadium, can be expressed as a linear combination, defined by *α*, of expected observed outcomes associated with those counties that did not open their stadiums. This vindicates our algorithmic approach, that is, the estimator presented in ref. 25, which exploits the low-rank structure in our observations to learn a linear predictive model.

Finally, we note an important aspect related to our implicit assumption of SUTVA. More specifically, SUTVA implicitly assumes that there is no interference between the different counties of interest. This is handled as described above via a careful selection of donor counties in which we discard any counties in which the data may be contaminated by a few fans who went to the games (as identified in the NFL attendance data).

### Methodology.

As discussed above, we use the estimator of ref. 25 to produce counterfactual estimates of what would have happened if the stadiums had not opened; here, we work through the methodology in more detail. Let *c*(*t*) represent the cumulative number of reported COVID-19 cases in the stadium county on day *t*. To find the number of cumulative cases in the synthetic county, we take a linear combination of case counts in the donor counties in the same state, where the coefficients are chosen to minimize the difference between the stadium county and its synthetic counterpart preintervention.

In using synthetic control with noisy data, it has been shown that robust results may be achieved by using a low rank estimate of the donor county matrix (16, 32). Namely, one can compute the SVD of Φ,

[5] $$\Phi =\left[\begin{array}{ccc} \phi_{11} & \phi_{12} & \ldots \\ \vdots & \ddots & \\ \phi_{T_{0}1} & & \phi_{T_{0}N} \end{array}\right]=\hat{U}\hat{S}{\hat{V}}^{T},$$

where column *i* represents the discretized time series of cumulative reported cases in donor county *i*. To develop a low rank representation of Φ, we define ${\hat{S}}_{\mu }$, which retains only the set of singular values above a threshold *μ* (with the remainder set to zero). In all of the data shown herein, we select *μ* such that the difference between synthetic and measured counties in the preintervention period is <1.0%. Our donor matrix is then approximated as

[6] $$E(\Phi )\approx \hat{\Phi }=\hat{U}{\hat{S}}_{\mu }{\hat{V}}^{T},$$

where $\hat{\Phi }$ is a rank *r* matrix whose columns represent the new set of basis functions that we will use to construct the synthetic control.

Combining the above, we arrive at the expression for the synthetic county,

[7] $$c_{\text{synth}}(t)=\sum_{i=1}^{N}{\hat{\alpha }}_{i}{\hat{\phi }}_{i}(t),$$

where the ${\hat{\alpha }}_{i}$s are selected by solving a least-squares problem that minimizes the difference between the measured cumulative number of cases in the stadium county and the synthetic county preintervention (we note that the spectral filtering step, followed by linear regression, is known as PCR).

Finally, we equip our estimates of the mean counterfactual outcome in the postintervention period with prediction intervals, as shown in the gray bands in Fig. 2,

[8] $$c(t)\in c_{\text{synth}}(t)\pm Z_{\text{CI}}\hat{\sigma }\sqrt{1+\langle \Phi (t),\hat{V}{\hat{S}}_{\mu }{}^{-2}{\hat{V}}^{T}\Phi (t)\rangle ,}$$

where $\hat{\sigma }$ is the SD of the difference between the stadium county and the synthetic county preintervention, and $Z_{\text{CI}}$ is the *Z* interval that defines the CI range. This suggests a test to determine whether or not estimates lie within the noise; that is, if the synthetic estimates lie outside of these confidence bands, then it may be the case that there is significant deviation due to the intervention.

### Latent Variable Comparison Metrics.

In order to compare relative distances in our reduced dimension framework with distances in other latent variable spaces, we first need to define an appropriate distance metric in our low-dimensional COVID-19 subspace. Distances between counties in this subspace can be represented by writing the measured case counts for each county as a linear combination of the basis vectors that arise from the SVD described above; if we retain *r* singular values, county *i* can be represented by a vector $C_{i}=(\beta_{i1},\ldots \beta_{ir})$ containing the coefficients *β_ij_* associated with each basis function. We then take the distance *d_ij_* between counties *i* and *j* to be

[9] $$d_{ij}=\left\|{\hat{C}}_{i}-{\hat{C}}_{j}\right\|,$$

where ${\hat{C}}_{i}$ is the unit vector aligned with the coefficient vector of the *i*th county $C_{i}$, and ||·|| represents an appropriate norm (here, we use the standard *L*^2^ norm).

This normalized distance is then used to compute the estimator *P_i_* in Eq. **2** and compared to relevant normalized metrics in other latent variable spaces. For example, political leaning is measured as the fraction of the county that voted Democratic normalized such that the “distance” between the most and least Democratic counties is one.^¶^

Finally, as a reference scale, we compute the expected “error” for a random estimator $P_{\text{rnd},i}$ which draws uniformly at random from the interval [0,1]. The distance, on average, between the measured $P_{\text{meas},i}$ and the random estimator is then given by

[10] $$\epsilon_{\text{rnd},i}\equiv E[\left|P_{\text{meas},i}-P_{\text{rnd}}\right|]=\int_{0}^{1}\left|P_{\text{meas},i}-P_{\text{rnd}}\right|\;dP_{\text{rnd}},$$

where E[·] represents the expected value. Computing the integral on the right-hand side, we find our expression for $\epsilon_{\text{rnd},i}$,

[11] $$\epsilon_{\text{rnd},i}=P_{\text{meas},i}^{2}-P_{\text{meas},i}+\frac{1}{2},$$

which is used in Eq. **3**.

## Data Availability

Previously published data were used for this work (19). All other study data are included in the article.

## Footnotes

^*^Note that these comments reflect the spring 2021 timing of the writing of this manuscript.

^†^An example of such a shock that occurred in Green Bay is presented in *Discussion*.

^‡^In addition, the synthetic counties accurately reflect the case counts in the months prior to the intervention ($R^{2}=0.99$ ) during which mitigation strategies were evolving.

^§^We note that such an assumption can be found in other works in the literature, including refs. 16 and 30.

^¶^Given that anomalously small counties may have unreliable COVID-19 statistics, we selected the most and least Democratic counties with at least 20,000 votes.
